# Applying nonlinear dynamics to the voice: a historical perspective

**DOI:** 10.1098/rstb.2024.0024

**Published:** 2025-04-03

**Authors:** W. Tecumseh Fitch

**Affiliations:** ^1^ Department of Behavioral and Cognitive Biology, University of Vienna, Vienna, Austria

**Keywords:** nonlinear dynamics, chaos theory, animal communication, speech science, vocal acoustics, bioacoustics

## Abstract

The recognition that nonlinear phenomena, including subharmonics, bifurcations and deterministic chaos, are present in human and animal vocalizations is a relatively recent one. I give a brief history of this revolution in our understanding of the voice, based on interviews with some of the key players and personal experience. Most of the key concepts and mathematical principles of nonlinear dynamics were already well worked out in the early 1980s. In the early 1990s, physicist Hanspeter Herzel and colleagues in Berlin recognized that these principles are applicable to the human voice, initially to baby cries. The physics and physiology underlying many of these nonlinear phenomena had remained mysterious up until then. This insight was later generalized to animal vocalizations. Nonlinear phenomena play a relatively peripheral role in most human vocal communication but are a common feature of many animal vocalizations. The broad recognition of the existence of nonlinear vocalizations, and the quantitative study of their production and perception, has now fuelled important and exciting advances in our understanding of animal communication. I concentrate on how the core concepts came into focus, and on their initial application to an ever-wider circle of call types and species, and end with a brief prospectus for the future.

This article is part of the theme issue ‘Nonlinear phenomena in vertebrate vocalizations: mechanisms and communicative functions’.

## 1. Introduction

Nonlinear dynamics and chaos theory represent one of the true scientific breakthroughs of twentieth-century science [1–5]. The key insight was that even quite simple mathematical systems, whose calculation is entirely deterministic, can nonetheless display highly complex and unpredictable behaviour depending on their starting point. We now know that this ‘sensitive dependence on initial conditions’ is quite typical in nonlinear systems, and applies in diverse fields ranging from physics and meteorology, through biology and medicine, to neuroscience and economics [4–6].

This realization has deep philosophical implications. It means that even in a fully deterministic universe, and with a detailed understanding of the laws of nature, scientists cannot accurately predict the behaviour of nonlinear systems beyond a strictly limited time period. Thus, even accepting (as Einstein quipped) that ‘God does not roll dice,’ Laplace’s dream that an all-knowing intelligence could perfectly predict the entire future is, for many real systems, false [7,8]. This unpredictability is completely independent of quantum indeterminacy, and the unpredictability inherent in nonlinear systems can be observed in macrolevel systems all around us, including weather, physiology or (as we will see) human and animal vocalizations.

An excellent history of the development of nonlinear dynamics is provided by Oestreicher [9]. Briefly, some of the key mathematical tools involved in nonlinear dynamics, such as phase diagrams, date back to the mathematical investigations of the three-body problem in celestial mechanics by Poincare in the nineteenth century. These diagrams, depicting what is now termed ‘phase space,’ eventually became one of the main visualization tools in this ‘new science’. But the modern field begins with the work of the meteorologist Edward Lorenz, who was using early programmable computers to do simulations of weather patterns [10]. Lorenz and colleagues found that seemingly trivial changes in starting conditions would lead, as the simulation progressed, to increasingly divergent overall outcomes of all parameters. Although the system of three coupled nonlinear equations making up Lorenz’s model is highly simplified (and from a mathematical viewpoint, simple), his results provided the crucial insight that even a totally deterministic system can show complex, unpredictable dynamics. Essentially, a superficially simple system in three dimensions can, under certain circumstances, exhibit unpredictable, chaotic behaviour.

**Figure 1.**
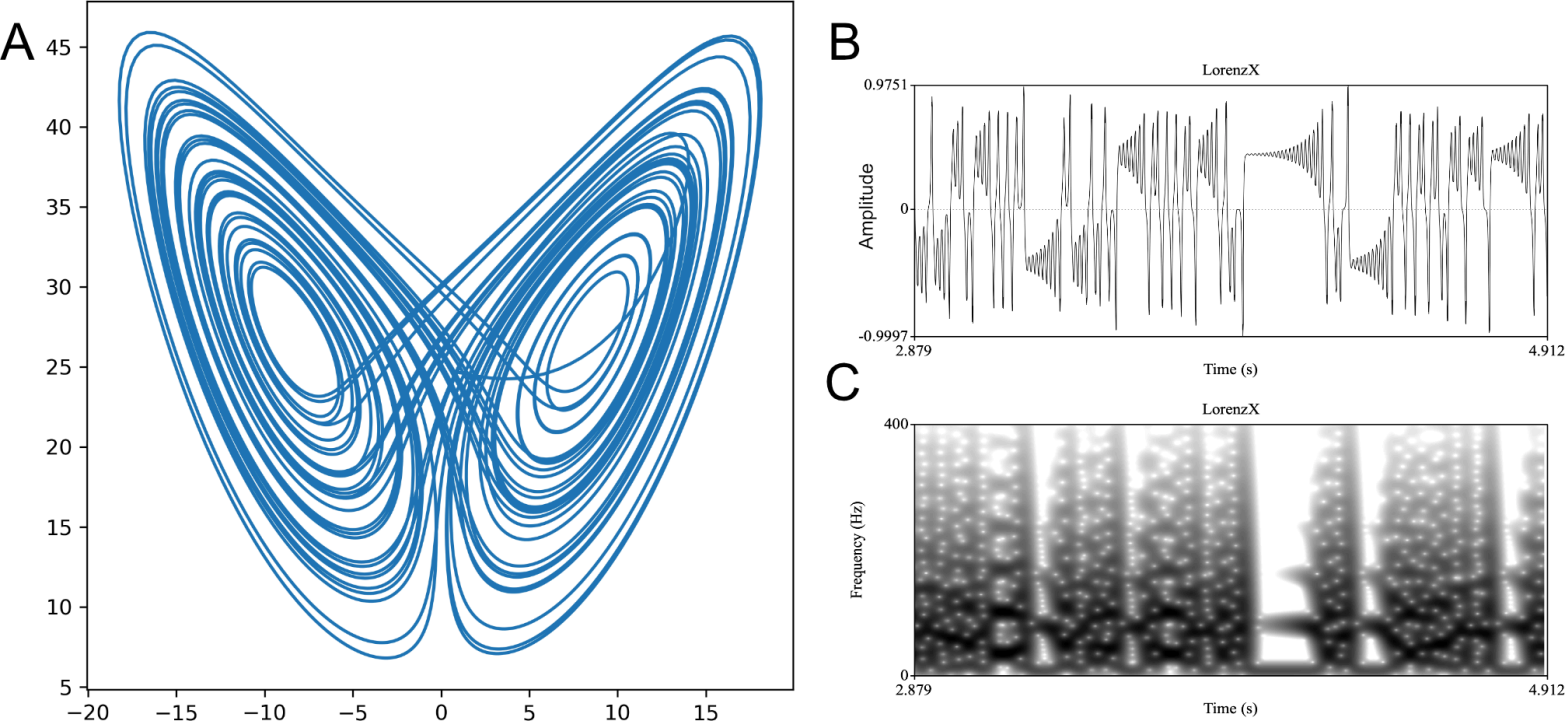
(A) The Lorenz attractor in two dimensions. (B,C) An example chaotic time series produced by one of the three variables of the Lorenz attractor, illustrating the irregular switching between ‘windows’ of periodicity and randomness that is common in chaotic signals ((B) time series, (C) spectrogram).

Later, in a famous paper, Lorenz posed the rhetorical question ‘Does the flap of a butterfly’s wings in Brazil set off a tornado in Texas?,’ thus giving a memorable name—‘the butterfly effect’—to the sensitive dependence on initial conditions revealed by his simulations [11]. Furthermore, Lorenz’s investigations showed that the dynamical system defined by his equations defined a very peculiar mathematical object in phase space, one that exhibited a mixture of periodicity at several different frequencies, with an unpredictable transition between these ‘windows’ of regularity. This is today termed a ‘Lorenz attractor’ and the general type of object it defines is known as a ‘strange attractor’ [12] (figure 1: Lorenz attractor).

Although Lorenz’s pioneering computer simulation work discovered the phenomenon of deterministic chaos, he did not use this term himself in this early work (instead he used the term ‘non-periodic flow’). Instead, the introduction of the term ‘chaos’ in its new technical sense is typically attributed (e.g. [4,9,13]) to James Yorke & Tien-Yien Li [14], who used the term ‘chaos’ in this context in 1975 (although I note that Robert May had used ‘chaos’ in the title and abstract of his publication in *Science* 1 year before: [15]). The widespread adoption of the term ‘chaos’ coincided with an explosion of interest in nonlinear dynamics in the mid-1970s, again driven partly by the increasing availability of computers, but now augmented by more formal mathematical investigations [14–19]. During this period, it became clearly recognized that the theory of nonlinear dynamics applies to a wide range of subject areas (including ecology, physiology, chemistry and electronics), and that certain universal properties of nonlinear systems can be discerned.

By the late 1970s, mathematical physicist Mitchell Feigenbaum, who was studying turbulence at Los Alamos laboratories, was able to show that the process of period-doubling bifurcations in any dynamical system follows a specific predictable pattern (the successive ratios occur at a constant interval of 4.669, now known as the Feigenbaum constant). Such a series can generate the broad-spectrum noise typical of turbulent systems in fluid dynamics, but it also characterizes a wide variety of other, seemingly unrelated, systems. Thus, despite the broad diversity of fields and phenomena that involve nonlinear dynamics, and the intrinsic quantitative unpredictability of these systems, they exhibit certain universal qualitative characteristics [12,14,20–23 ]. At this point, it became increasingly clear that, rather than a diverse set of tools applied to a potpourri of disparate phenomena, nonlinear dynamics actually offers a unifying theory and a set of mathematical principles covering a huge range of systems. The term ‘chaos theory’ became increasingly applied to this body of research [3,24], and Gleick’s popular science book ‘*Chaos: making a new science*’ brought this theory and its history to a wide audience of both scientists and laypeople.

## 2. Chaos theory and biology

Biologists played an important role in the early history of chaos theory. Indeed, theoretical biologist Robert May is counted as one of its founding fathers, due to two early high-visibility papers published in *Science* and *Nature* [15,17] which explored simple systems of equations related to population growth in ecology (such as the Lotka–Volterra equations and the logistic equation). May’s analyses showed that, in populations of several species that are coupled in some way (e.g. as predator and prey, or diseases and hosts), wildly unpredictable dynamics can result for certain parameter values, even in fully deterministic systems [25]. Although May sketched some potential applications to natural populations (e.g. control of insect pests), his goal was to ‘provoke applications in yet other fields’ and he ended with a plea that more biologists should become aware of nonlinear dynamics.

May’s call was quickly taken up: in the following year, physiologists Mackey and Glass demonstrated the applicability of nonlinear dynamics to human physiology, and how it specifically applies to the coupled oscillators of breathing and heart rate (specifically, a potentially life-threatening medical phenomenon known as Cheyne–Stokes breathing) or to blood cell production in leukaemia patients [16]. These subfields of physiology, particularly cardiac physiology, remain core areas where the application of chaos theory is widely recognized as both an important theoretical advance, and also practically necessary to understand the underlying dynamics of certain forms of heart irregularity. This paper also introduced the term ‘dynamical diseases’ to indicate situations in which an ordinary, non-pathological biological control system can, with particular parameter settings, become unpredictable and chaotic.

Perhaps surprisingly, given the highly nonlinear nature of neurons, it has taken much longer for the theory of nonlinear dynamics to find applications in neuroscience (e.g. [26]). Although it remains a topic of interest [27–29], successful applications of chaos theory in the brain remain rare. This is probably because, although the brain is certainly a highly nonlinear system, the dimensionality of neuronal systems is so high that in most cases, it is more profitable and revealing to analyse neural dynamics with stochastic models (e.g. probability distributions) rather than systems of coupled nonlinear differential equations.

There is an important lesson here: the crucial contributions of chaos theory come from its ability to make quantitative predictions about dynamics leading up to the transition to chaos, including bifurcations, but this is only really possible for relatively low-dimensional systems [21]. Once the system has transitioned to full-blown chaos (e.g. full turbulence in a fluid system), the theory ceases to have more than a rough, heuristic value and the more familiar stochastic models of statistics become more useful. This insight is important in understanding applications of nonlinear dynamics to the voice, to which we now turn.

## 3. Nonlinear dynamics in the human voice

‘*Chance favours the prepared mind*’—Louis Pasteur

By the mid-1980s, chaos theory was a well-developed discipline [9]. Gleick’s popular book had helped the excitement and promise of this theory reach a wide audience, and textbooks were being written on the topic [2,3,30]. The journal *Science* published a six-article series on chaos theory [24], and the interest of biologists in fields like physiology or ecology was already secured. However, as discussed above, the productive application of nonlinear dynamics to biological phenomena requires relatively simple, low-dimensional systems and such systems are only moderately common in biology. Furthermore, physicists were used to laboratory experiments with very stable stationary systems, where large amounts of data could be used to test and refine simple models, whereas most biological systems are relatively unstable and non-stationary due to factors like homeostasis, nutrition, climate, etc. Thus, applications in biology, although well-known [2,15,17], potentially appeared to be somewhat limited. Thus, the application of chaos theory to vocal production, where the required conditions of simplicity and low dimensionality are often met, was a major step forward, not just for voice science but for chaos theory more generally.

The key insight was that certain phenomena in vocal production, particularly screams and similar harsh, apparently atonal sounds, represent chaos in the acoustic domain. The history of this advance is the topic of the remainder of this paper, and it is largely due to the insights of one scientist—Hanspeter Herzel, at the Humboldt University in Berlin—and his close colleagues. My main source for the following information comes from a two-part recorded interview with Herzel conducted in 2023, along with discussions and formal interviews with other experts in this field (see ‘Acknowledgements’).

Hanspeter Herzel was born in Güstrow, Germany in 1957, and grew up behind the Iron Curtain in what was then East Germany, in Berlin. He was initially interested in mathematics, but always had a keen interest in combining models with data, and thus opted to study physics. He was trained in statistical physics in the laboratory of Werner Ebeling at Humboldt University Berlin. In the late 1980s, Ebeling ran a very flexible laboratory, where many interesting ‘hot topics’ in contemporary science were being investigated by different groups, including neural networks, pattern formation and of course chaos theory. Herzel’s work in Ebeling’s laboratory involved applying tools of nonlinear dynamics to understanding stochastic sequences and chemical reactions (e.g. [31]), and understanding the effects of noise and thermodynamics by calculating entropy and other measures. However, the excitement of chaos theory had also led to a wealth of speculation, and there were claims of chaos popping up in many fields. This led to a degree of scepticism, and an increasing awareness that it is not easy to empirically demonstrate chaos in real systems. Thus, Herzel was eager to find new data-driven applications in which the physical and mathematical tools of nonlinear dynamics were truly applicable to real-world systems.

Herzel began teaching about nonlinear dynamics in 1985, and was very familiar with Schuster’s early textbook on deterministic chaos [30]. In Herzel’s teaching, he used Schuster’s textbook, and one of the opening plates in this book is a spectrogram illustrating bifurcations and period-doubling through subharmonics to chaos (plate VI, [30]). From teaching this material, Herzel became familiar with research in acoustics from studies in Werner Lauterborn’s experimental physics group in Göttingen on the nonlinear dynamics of acoustic cavitation [32,33]. From this, Herzel was already familiar with acoustics and spectrograms, and knew what nonlinear phenomena look like in spectrographic representations.

Herzel dates his realization that there is chaos in the voice to a meeting with fellow PhD student Kathleen Wermke at a computing facility in Berlin in late 1986. Wermke had just completed her PhD thesis on human infant cry in Berlin [34], and her thesis contained printouts of spectrograms of seemingly tonal infant cries that rapidly transitioned into harsh, atonal cries. When Wermke showed Herzel these spectrograms, Herzel immediately recognized that they clearly illustrated bifurcations into deterministic chaos. Working together with Wermke and her mentor, the mathematician Werner Mende, Herzel went on to perform detailed analyses of such cries that culminated in April 1990 in the landmark publication in this field ‘Bifurcations and chaos in newborn infant cries’ [35]. This paper discovered and documented low-dimensional chaos in infant cries, and concluded that chaos theory may provide ‘a new approach to the understanding of some aspects of speech production’—in retrospect a prescient but overly modest statement.

Herzel realized that the voice potentially represented a domain where nonlinear tools were both appropriate, currently unappreciated, and of potential practical relevance. In 1987, after finishing his 1986 PhD in statistical physics (for which he received the Humboldt prize), he embarked upon a detailed reading of the speech science and vocal physiology literature, and he quickly came to understand that the two vocal folds represent coupled nonlinear oscillators of relatively low dimensionality: a perfect domain for the application of chaos theory. The next step was to develop computer models to allow more thorough exploration of dynamics, calculation of bifurcation diagrams, etc. During a sabbatical at the Technical University of Denmark, he proposed to Carsten Knudsen to use software running there to do nonlinear analysis of vocal fold models, but was told that the existing models [36] were too complex for this purpose. Thus, once he felt he understood the ‘standard’ Ishizaka and Flanagan model well, Herzel developed a computer model of the vocal folds that simplified this model enough to run on an Atari computer [37]. This simplification allowed him and his colleagues to rigorously demonstrate that the full suite of nonlinear dynamical behaviour occurs in a realistic physical model of the vibrating vocal folds, including bifurcations, period doubling, subharmonics and chaos [38,39].

Simultaneously, Herzel began working with researchers at the Charité hospital in Berlin on adult voice patients, and it became clear that various nonlinear phenomena can be readily observed in pathological voices, e.g. in patients where one vocal fold was paralysed. This was consistent with the computer modelling results. Although nonlinearities could be obtained with symmetric models, the full suite of nonlinear phenomena was most easily observed when the two vocal folds were asymmetric (e.g. with decreased tension in the fold on one side). Later, such asymmetry was directly demonstrated, using high-speed video and endoscopic visualization of the larynx, in both a vocal paralysis patient and a normal woman with asymmetric vocal folds who could sing, simultaneously, on two separate pitches [40]. Thus, by 1991, it was already clear to Herzel and a set of close colleagues in Berlin that nonlinear dynamics applies to the human voice, both in theory and in natural infant cries and recordings of voice patients [38].

Herzel then decided to contact international speech researchers around the world to see if others had reached this same conclusion, and sent letters to a number of top speech researchers. He got three replies. Two said, ‘Contact Ingo Titze, he might know’. The third came from Titze himself, and immediately invited Herzel to come to the USA, and to co-organize a workshop on the topic of nonlinear phenomena in the voice, at the Voice Physiology Conference in Denver in 1992. This workshop, co-organized with Osama Fujimura, was probably the first time that a large international group of voice researchers came together to discuss the topic, and recognized the importance of methods from nonlinear dynamics to the study of the human voice (particularly in vocal pathology). This workshop culminated in a book chapter titled ‘Evidence of chaos in vocal fold vibration’ [41] in an edited volume on vocal fold physiology.

The Berlin Wall fell in November 1989, meaning that East German citizens like Herzel were finally free to travel. A Heisenberg Fellowship to Herzel allowed him to put this freedom to use: he travelled 2−3 times per year to the USA, frequently visiting Titze’s lab in Iowa, as well as other voice and speech labs throughout the USA and Europe. Herzel embarked on an extensive study of the methods of voice science, and familiarized himself with excised larynx experiments during the next 3 years. In multiple collaborations, he and colleagues both broadened the scope and deepened the understanding of nonlinear phenomena in the human voice. It became increasingly clear that the vibrating vocal folds represent two coupled nonlinear oscillators, exhibit the full suite of nonlinear phenomena, and constitute an ideal platform to observe and understand low-dimensional chaos in a real physical system.

However, it also became clear that during ‘normal’ speech and singing, the voice of healthy human adults typically exhibits stable periodicity, avoiding nonlinear phenomena like subharmonics and chaos. Most adult human communication sounds thus involve highly predictable, periodic oscillations, although nonlinear phenomena can be observed in voice patients, or in certain non-verbal utterances like screaming, or laughter in human adults [42]. Certain non-classical singing styles also feature nonlinear phenomena [43,44]. Despite such exceptions, it seemed perhaps that nonlinear phenomena in the voice were something of a niche subject, with applicability mainly to vocal disorders and/or non-verbal communication. This estimate of the importance of nonlinear phenomena to the voice was soon to change, with the realization that the same principles and analysis methods are clearly applicable in non-human species as well, and in many cases in animal communication, the phenomena themselves are extremely common.

## 4. Nonlinear dynamics in vocalizations of non-human animals

Up until 1996, the application of nonlinear dynamics to the voice was limited to the human voice, and particularly to infant cries and pathological voices. This changed when Herzel accepted a position in the Biology Department at Humboldt University. In one of his early talks in that position, he presented the core theory and methods of nonlinear dynamics, along with their applications to the human voice, and was surprised that the biologists in the audience did not seem particularly excited. The main thing that seemed to catch the interest of many of these biologists was the idea that animals might use subharmonics to acoustically exaggerate the impression of body size conveyed by their calls.

Fortunately, however, these presentations excited two curious PhD students, Inka Wilden and Tobias Riede, who had noticed frequent strange phenomena in recordings of animal vocalizations. After meeting Herzel, they asked their supervisor Professor Günter Tembrock to allow them to work on nonlinear phenomena in animals. Tembrock had amassed a large body of animal recordings, and an exploration of this bioacoustic archive (the Tierstimmenarchiv Berlin) suggested that nonlinear phenomena might be more prevalent in animal vocalizations than in those of adult humans. This exploration culminated in the first published applications of nonlinear dynamics to mammal voices, led by Inka Wilden [45] and Tobias Riede [46]. This was quickly followed by an independent application of nonlinear dynamics to bird vocalizations led by Michale Fee at Bell Labs [47]. It began to seem possible that nonlinear phenomena play a more central role in animal vocalizations than in human speech (cf. [48]).

I first met Hanspeter Herzel in 1996, when I was a post-doc at the MIT/Harvard Speech and Hearing Sciences programme. I was aiming to adapt methods developed to understand the human voice to studying animal vocal production, and frequently attended lectures at MIT’s Research Laboratory of Electronics speech science group, led by Kenneth Stevens. Herzel was invited to present his research on nonlinear dynamics of the human voice, and in attendance were Ken Stevens, Joe Perkell and Stephanie Shattuck-Hufnagel—some of the top experts on acoustic phonetics in the world at the time (e.g. [49,50]). I went to see Herzel’s talk together with another animal communication post-doc at Harvard, Julia Fischer. Both of us had been working with macaque vocalizations, and were already familiar with the so-called ‘noisy’ calls that are relatively common in primate vocal repertoires. The summer before, I had also worked on a project attempting to synthesize human infant distress cries, which possessed both clear tonal components and an overlain or interspersed ‘noisy’ component. Using computer synthesis, I tried to reproduce this phenomenon by combining normal infant cries with synthesized white noise. The result was a complete failure: the synthesized sounds possessed nothing of the harsh, annoying urgency of a real infant distress cry. Humbled and disappointed, I admitted I had no idea how such sounds were produced, and thus how to synthesize them, and abandoned the project.

**Figure 2.**
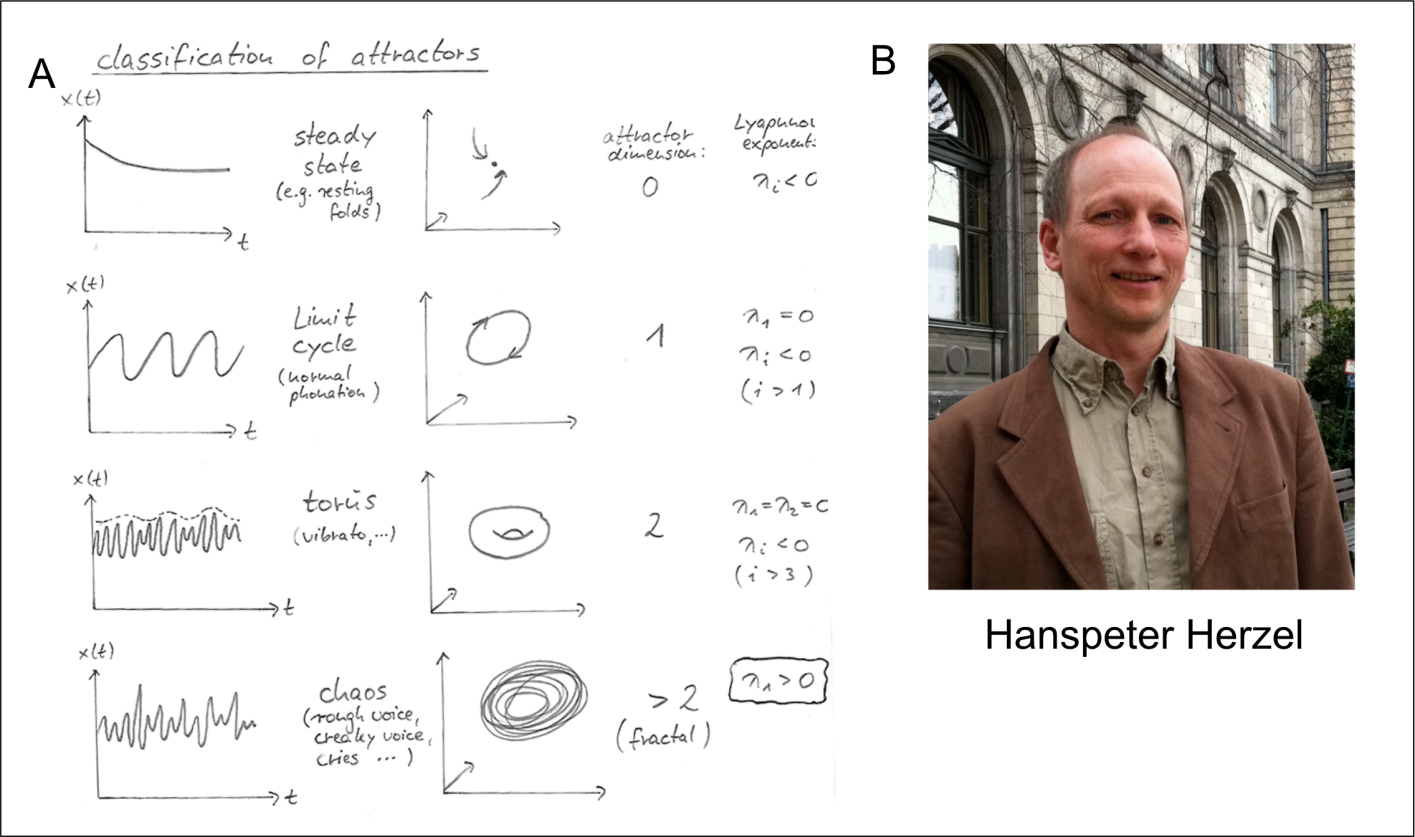
Hanspeter Herzel (B) and one of his characteristic hand-drawn lecture transparencies (A) introducing the main ideas of nonlinear dynamics and chaos theory on a single sheet.

Herzel presented most of his MIT talk on an overhead projector, starting with a blank transparency. He carefully introduced the key concepts of nonlinear dynamics, drawing oscillations, phase plots and bifurcation diagrams on the transparency as he went (see figure 2 for an example), and often imitating the key nonlinear phenomena using his own voice. By the time he had reached the bottom of the first transparency, he had explained limit cycles, bifurcations, subharmonics and chaos and shown (and vocally illustrated) how they apply to the human voice (figure 2). Julia and I were fascinated and amazed—these exciting concepts seemed obviously applicable to primate vocalizations! Although I had read Gleick’s popular book ‘*Chaos*’ long before, and so was familiar with general notions of chaos theory in physics, and with some of the underlying mathematics, I had no idea that these concepts were applicable to either human or animal voices until Herzel’s presentation. This talk thus had a momentous effect on my thinking about animal vocal production—it clarified issues that had been bothering me for years, and naturally led to a host of other exciting questions. Julia and I introduced ourselves to Herzel after his talk, and initiated an email correspondence. He invited me to give a talk in Berlin the following January, and we began an intensive collaboration applying nonlinear dynamics to animal vocalizations.

I returned to Berlin several times, including for a major voice physiology conference in March 1999, organized by Herzel, that was the first time that the human vocal fold experts like Ingo Titze were brought together with top bioacousticians interested in animal vocal production (including Roderick Suthers, the doyen of the field). At this point, in the late 1990s, it was becoming clear to researchers interested in the biophysics of animal vocal production that nonlinear dynamics provided a promising way forward. My collaboration with Herzel over 1997−2002 culminated in our review article ‘Calls out of chaos,’ published in *Animal Behaviour*, which introduced the concepts of nonlinear dynamics of the voice to a significant number of researchers on animal communication who had no previous background in physics or vocal physiology [51]. In that paper, we presented data from my field recordings of rhesus macaques demonstrating that, far from being a fringe or pathological phenomenon, nonlinear phenomena occur in a sizeable proportion of the calls emitted by normal macaques (in my sample of 467 calls, about 30% overall exhibit nonlinear phenomena, but this proportion rises to 40% for subadults and a whopping 60% for females). This demonstrated that no one hoping to understand the full range of communication in macaques (and by extension other primates) could afford to ignore nonlinear phenomena, and suggested that in fact this new body of theory and methods might hold the key to unlocking long-ignored but crucial components of animal communication systems.

From about 2002 onward, the animal literature on nonlinear dynamics in vocalization exploded (cf. [48]), and nonlinear phenomena have now been documented in a wide variety of animal vocalizations, including insects, frogs, crocodilians, birds and many mammals including primates, rodents, carnivorans, cetaceans, manatees and elephants [52–60]. This indicates that such phenomena are present in all of the main sound-producing taxa, and suggests that they are relatively common components of vertebrate vocal repertoires. Thus, the application of chaos theory to animal vocalizations represented a major step forward in bioacoustics, and catapulted our understanding of previously mysterious vocal phenomena to an entirely new level.

## 5. Were vocal nonlinear phenomena ‘in the air’?

As emphasized above, the realization of the existence and significance of nonlinear phenomena to vocal production was largely the work of one scientist, Hanspeter Herzel, and his close colleagues. Werner Mende (who acted as informal supervisor to Kathleen Wermke) suspected bifurcations and chaos in infant cry recordings, and this suspicion fuelled the work with Herzel that led to their 1990 publication [35]. However, there are several indications that the idea of ‘chaos in the voice’ may already have been ‘in the air’ in the late 1980s. The clearest indication is the publication in 1989 (book) and 1990 (article) of an analysis by the Polish physicist and mechanical engineer Jan Awrejcewicz of bifurcation analyses of a set of nonlinear equations modelling the vocal folds [61,62]. This was theoretical work, continuing Awrejcewicz’s work on nonlinear dynamics of a wide variety of physical systems, and presented no actual data from voices. Furthermore, the focus was on methods for generating bifurcation diagrams, rather than any apparent interest in vocal behaviour. Similarly, Ronald Baken published a paper in 1990, as had Pickover & Khorasani [63], using fractal-based methods to analyse voice irregularity [64]. These publications clearly presage the usage of more insightful dynamical methods introduced by Herzel at about the same time, and clearly indicate that the general notion of applying chaos theory to the voice was not an isolated event.

An anecdote told by Hanspeter Herzel provides a second indication that vocal chaos was an idea whose time had come. During one of his many trips to the USA in the early 1990s, he visited the laboratory of Fariborz Alipour and Ronald Scherer, who had been performing excised larynx experiments using dog larynges in the previous years. Herzel was watching videos of these experiments, and in one of them, the dog larynx was producing irregular vibrations. Scherer’s voice could be heard in the background saying ‘this is a lousy larynx,’ and the experiment on that particular specimen was abandoned rapidly. However, in the laboratory notebook recording those experiments, on the following day, Scherer had taped in a figure of a bifurcation diagram taken from Gleick’s ‘*Chaos*’ book. This suggests that Scherer suspected that chaos theory was relevant to the voice, at least in certain isolated cases, although he credits Herzel with the demonstration that this is the case.

## 6. The present and future of nonlinear dynamics and chaos theory in animal vocalizations

Despite my focus here on history, I will end with a brief look at recent advances with an eye towards the future of this field. Although the last 25 years have seen major advances in the application of nonlinear dynamics to animal vocalizations, these are still early days. Much past work simply documents that nonlinear phenomena occur in a given species, sometimes with a tally of how common different forms are. But multiple fundamental questions remain to be answered concerning mechanisms, measurement and evolution.

Starting with mechanisms, recent work on primates suggests that there are specific anatomical modifications to vocal anatomy that increase the likelihood of nonlinear phenomena. In particular, the vocal membranes observed in many mammal species, including most primates, appear to increase the instability of vocal fold vibrations and thus their propensity to enter subharmonic and/or chaotic regimes [65–67]. Vocal membranes are thin ribbon-like extensions of the upper lip of the vocal folds that appear to both support high-frequency phonation and destabilize the lower frequency vibrations of the more massive body of the vocal fold. Intriguingly, these vocal membranes are found in some form in most primates including chimpanzees, but have been lost in humans, suggesting selection for a more stable voice in our species [67]. There are many other poorly understood aspects of comparative vocal anatomy, such as air sacs, that could also potentially have a destabilizing effect [68–70].

Vibrating laryngeal tissues other than the vocal folds provide another important understudied factor in nonlinear vocal phenomena, in both humans and other animals [51]. For example, the ventricular folds (a set of additional folds, similar but anterior to the vocal folds, sometimes called ‘false vocal folds’) can be engaged in phonation. The ventricular folds constitute independent oscillators that, especially when coupled to the vocal folds, can lead to nonlinear phenomena [43,71]. The ventricular folds have recently been shown to play a role in ‘growl’ phonation in bats [72] along with certain singing styles (e.g. screams and growls in death metal singing). Ventricular folds are present in primates, where they may also bear vocal membranes [66], and in some other mammal clades, but are absent in other species [73]. It seems likely that some of the variation in nonlinear phenomena across mammalian vocal repertoires may be tied to the presence or absence of these understudied ventricular folds. More generally, nonlinear dynamics may hold the key to unlocking the function of hitherto mysterious anatomical features of the vocal apparatus across many other clades (e.g. [60]).

Turning to issues of measurement, there are multiple open questions about how to best quantify nonlinear phenomena [74]. The ‘classical’ measures of nonlinearity in chaos theory are Lyapunov exponents and fractal dimensionality [21,33], but properly calculating these measures requires long time-series during which the key control parameters (such as vocal fold tension or driving air pressure) are constant [21,75]. Unfortunately, this criterion is rarely met in natural animal vocalizations, where control parameters are typically changing constantly. Thus, with the exception of some insect vocalizations (or excised larynx experiments, see below), Lyapunov exponents can rarely be properly calculated for animal vocalizations. This has led to a considerable number of proposed alternative measures, such as Tokuda and colleagues' low-dimensional nonlinearity measure [75], and visualization methods, such as visual recurrence analysis [76] or Herbst and colleagues' ‘phasegrams’ [77]. Also widely used is the harmonics-to-noise ratio (HNR). HNR provides a measure of ‘hoarseness’ but requires a stable and accurately measured fundamental frequency to produce meaningful results, rendering it of limited value in quantifying nonlinear phenomena like chaos [78]. Although many of these existing methods are now implemented in free software and thus easily available (e.g. [76,79,80]), there is no current consensus about the best measure (or set of measures) for quantifying chaos and other nonlinear phenomena in the voice.

An exciting experimental domain where both stable parameter values and long time-series can be obtained is provided by excised larynx research. These *ex vivo* experiments involve mounting the excised vocal organ (larynx or syrinx) in an apparatus that provides a controlled flow of warm, moist air between the vibrating structures, and allows precisely controlled pressure and vocal fold tension [47,81,82]. In such experiments, it is possible to have long stretches of vocalization with constant control parameters, alleviating some of the measurement problems discussed above. Excised larynx research also makes quantitative comparisons between species possible (e.g. [83]) or between data and models [47], allowing questions about the relative instability and propensity for chaos in species with different vocal anatomy to be rigorously addressed. Although excised larynx experiments have been performed in a variety of species, including humans and other primates, frogs, rodents, deer, dogs, elephants and whales [83–88], the potential of this method for addressing fundamental mechanistic issues in nonlinear vocal dynamics, in a comparative context, still remains mostly untapped.

The topics above concern the mechanistic basis and quantitative measurement of nonlinear vocal phenomena. However, there are many open questions about their ultimate evolutionary function [51] that remain unanswered today. For example, it has been proposed that the intrinsic unpredictability of chaos makes chaotic vocalizations, such as screams, difficult to habituate to and thus difficult to ignore [89]. Although this hypothesis has been explored in humans (e.g. [90,91]), and is consistent with some animal data [56], it has only been directly tested in one animal species [92], who found that meerkats indeed take longer to habituate to vocalizations incorporating nonlinear phenomena. The widespread occurrence of screams in vertebrate vocalizations (including in mammals, birds and frogs) makes this hypothesis ripe for further comparative perceptual testing. Other possible adaptive functions of nonlinear phenomena have received even less attention. For example, the proposal mentioned earlier that subharmonics may provide an easy way to lower apparent fundamental frequency, or the possibility that screams may highlight or obscure the vocal tract transfer function [51], remain tantalizingly unexplored.

Finally, the perception of nonlinear phenomena remains less explored than their production, with most existing work performed with humans (cf. [78], but see [56,60]). Although white noise and chaos sound very different to the human ear, in one playback study, no significant difference was found between responses to white noise and two forms of synthesized chaos by listeners of two bird species [93]. This suggests that different species may vary widely in their sensitivity to different types of nonlinearities, and again opens a wide and fertile field for future experimentation.

In general, one might suggest that the study of nonlinear dynamics in animal vocalization has been in its ‘natural history’ stage for the last two decades: documenting the existence of nonlinear phenomena across a wide range of species, and beginning to understand the mechanisms underlying them. But when we turn to the perception, measurement, and in particular adaptive function of these phenomena, there are currently many more open questions and plausible hypotheses than data-based answers. I suggest that this field is now ready to move beyond this initial stage, and launch into exciting topics of physiology, perception and adaptive function, and that nonlinear phenomena can take their rightful place as core components in animal communication research.

## 7. Conclusion

The application of the theory and methods of nonlinear dynamics to vocalizations, despite its relative recency, has allowed students of both animal communication and the human voice to understand a host of phenomena and vocalization types that were previously mysterious. The theory of nonlinear dynamics in general has a long history, stretching back about 60 years, and was well-established by the late 1970s. But I have attempted to document here how exciting new insights leading to the application of this theory to the voice emerged during a relatively short time period in the late 1980s and early 1990s, in a small group of scientists in Berlin led by physicist Hanspeter Herzel. The history since then has been one of ever-widening scope, and widespread realization that nonlinear phenomena are prominent aspects of vocal communication in a broad range of species. There is no sign that this exhilarating process has reached an end, and all evidence points toward continuing advances in multiple directions, and further detailed exploration of the role of nonlinear phenomena in the evolution of communication.

## Data Availability

This article has no additional data.
